# Modeling Endothelialized Hepatic Tumor Microtissues for Drug Screening

**DOI:** 10.1002/advs.202002002

**Published:** 2020-09-21

**Authors:** Ying Wang, Ranjith Kumar Kankala, Jianting Zhang, Liuzhi Hao, Kai Zhu, Shibin Wang, Yu Shrike Zhang, Aizheng Chen

**Affiliations:** ^1^ Institute of Biomaterials and Tissue Engineering Huaqiao University Xiamen 361021 P. R. China; ^2^ Fujian Provincial Key Laboratory of Biochemical Technology Huaqiao University Xiamen 361021 P. R. China; ^3^ Department of Cardiac Surgery Zhongshan Hospital Fudan University Shanghai 200032 P. R. China; ^4^ Division of Engineering in Medicine Brigham and Women's Hospital Department of Medicine Harvard Medical School Cambridge MA 02139 USA

**Keywords:** anticancer, drug screening, hepatic models, multicellular tumor aggregates, vascularization

## Abstract

Compared to various traditional 2D approaches, the scaffold‐based 3D tumor models have emerged as an effective strategy to investigate the complex mechanisms behind cancer progression and responses to drug treatments, by providing biomimetic extracellular matrix and stromal‐like microenvironments including the vascular elements. Herein, the development of a 3D endothelialized hepatic tumor microtissue model based on the fusion of multicellular aggregates of human hepatocellular carcinoma cells and human umbilical vein endothelial cells cocultured in poly(lactic‐*co*‐glycolic acid)‐based porous microspheres (PLGA PMs) is reported. In contrast to the conventional 2D culture, the cells within the PLGA PMs exhibit significantly higher half‐maximal inhibitory concentration values against anticancer drugs, including doxorubicin and cisplatin. Furthermore, the feasibility of coculturing other cell types, such as fibroblasts (L929) and HepG2 cells, is investigated. Together, the findings emphasize the significance of engineered 3D hepatic tumor microtissue models using PLGA PM‐based multicellular aggregates for drug screening applications.

## Graphical abstract

The development of a 3D endothelialized hepatic tumor microtissue model based on the fusion of multicellular aggregates of human hepatocellular carcinoma cells and human umbilical vein endothelial cells cocultured in poly(lactic‐*co*‐glycolic acid)‐based porous microspheres (PLGA PMs) is reported. The significance of engineered 3D hepatic microtissue models using PLGA PM‐based multicellular aggregates for drug screening applications is demonstrated.

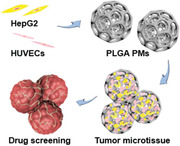

## 1 Introduction

Over the past few decades, enormous efforts have been dedicated to exploring diverse effective preclinical screening methods toward explicitly demonstrating the pharmacological as well as toxicological attributes of various therapeutic drugs. Despite the advantages, the widely applied, traditional cell monolayer‐based 2D approach often suffers from shortcomings in recapitulating the highly complex, natural extracellular matrix (ECM)‐like microenvironments, such as cell–cell as well as cell–matrix interactions.^[^ ^1^ ^]^ To overcome these limitations and to achieve better replication of the in vivo tumor characteristics, 3D tumor models based on cell aggregates have emerged as a promising alternative in mimicking the sophisticated spatial arrangements of the cells for predicting the efficacy of and resistance against antitumor agents toward drug evaluation and cancer research.^[^ ^1^, ^2^ ^]^

To this end, numerous fabrication strategies have been reported for generating cell aggregates, such as cell sheet engineering,^[^ ^3^ ^]^ hanging drop method,^[^ ^4^ ^]^ and microwell plate culture.^[^ ^5^ ^]^ More recently, tremendous progress has evidenced the applications of 3D scaffold‐based models toward tumor tissue engineering and diagnostics.^[^ ^6^ ^]^ As reported, these 3D scaffolds reflect a more accurate tumor microenvironment compared to the traditional 2D platforms and 3D spheroid cultures.^[^ ^7^ ^]^ Among various scaffolds used for 3D cell cultures, polymeric porous architectures offer advantages in terms of cellular interactions and ECM remolding. Mooney and co‐workers first reported the fabrication of a 3D human tumor model based on poly(lactic‐co‐glycolic acid) (PLGA) porous scaffolds for culturing oral squamous cell carcinoma (OSCC‐3) cells, which reinforced the necessity of recapitulating tumor ECM‐mimic microenvironment.^[^ ^8^ ^]^ In addition, these porous architectures facilitated cell attachment attributing to the internal pores with interconnecting windows. For instance, gelatin‐based porous microscaffolds effectively promoted cell growth and metabolic activity by inducing deposition of a higher amount of extracellular proteins compared to multicellular spheroids, representing a biometric system to mimic the stromal element of the tumor tissues in vitro.^[^ ^9^ ^]^ In another case, Menon and colleagues reported the construction of a lung tumor model using PLGA‐based porous microparticles encapsulated with A549 lung adenocarcinoma cells.^[^^]^ Notably, these cell‐laden composites showed greater drug resistance compared to conventional cell monolayers. Although some of these prevailing models have proven to be effective in mimicking the complex compositions of the tumors, there is still a strong need to improve the efficacy and cell–cell interactions of 3D scaffold‐based microarchitectures. In our previous study, PLGA porous microparticles (PMs) were fabricated using the microfluidic technology for cell delivery.^[^ ^11^ ^]^ Owing to their high porosity and biocompatible attributes, these microarchitectures have shown great potential in the fabrication of cell‐laden PMs for various applications in vitro.^[^ ^12^ ^]^

With regard to the complex architectural organizations of multiple cell types in the native tumor tissues, coculturing nonparenchymal cells with tumor cells can enhance cell–matrix interactions, which regulate the growth, proliferation, and metastasis of tumor cells.^[^ ^13^ ^]^ In this regard, numerous advancements have been evidenced based on not only the formation of tumor spheroids but also the effects of vascular system on the growth of tumors.^[^ ^14^ ^]^ In the native tumor tissues, the cells can directly obtain the tumor‐inducible factors via the paracrine effects of endothelial cells (ECs). Conversely, the angiogenic growth factors are secreted to induce EC sprouting into tumor tissues and renew the vessels for nutrient transport during tumor formation.^[^ ^15^ ^]^ In the absence of vascular networks, the nutrient supply and metabolite excretion rely on the mere physical diffusion, which severely limits the growth of tumor cells and the possibility of tumor cell migration. Coculturing of ECs with tumor cells is the most direct approach to establish the vascular functions toward the construction of tumor tissue models.^[^ ^2b^ ^]^ For instance, bioengineered liver (steatosis) spheroids were fabricated based on coculturing the human umbilical vein ECs (HUVECs) with human hepatocellular carcinoma (HepG2) cells.^[^ ^16^ ^]^ In another example, ECs cocultured with HepG2 hepatocellular carcinoma cells in a 3D culture system resulted in their differentiation to tubular networks, leading to the upregulation of essential signaling molecules in the volumetric tumor model.^[^ ^17^ ^]^ Furthermore, in our previous work, the microchannels cultured with lymphatic microvascular ECs were embedded into hydrogels containing breast tumor cells, resulting in the development of a human breast cancer lymphangiogenesis model.^[^ ^18^ ^]^ Together, mimicking the interactions between ECs and tumor cells in vitro may help in investigating tumor initiation, cancer pathologies, and therapeutics development.

**Figure 1:**
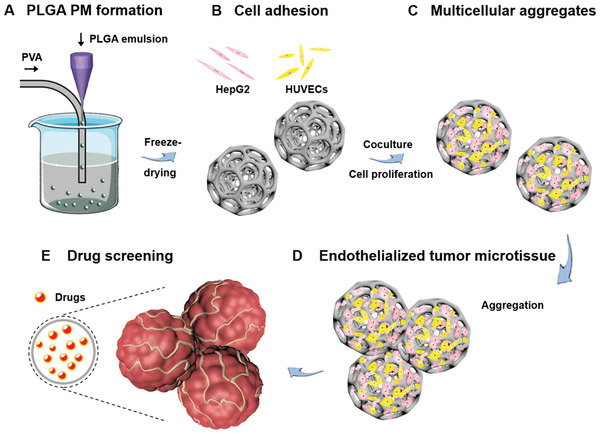
Outline of the method. Schematic illustrations showing the fabrication of a multicellular aggregate‐enabled tumor model based on PLGA PMs and potential applications in constructing tumor microtissues in drug screening. A) Generation of PLGA PMs using the microfluidic technology. B,C) Adhesion and proliferation of HUVECs and HepG2 cells in the PLGA PMs leading to formation of endothelialized multicellular tumor aggregates. D) Construction of the endothelialized tumor microtissue through further interactions of individual multicellular tumor aggregates. E) The chemotherapeutic drugs, DOX and CIS, were employed as model drugs to carry out the anticancer drug assessments of the endothelialized tumor microtissues.

Inspired by these works, here we demonstrate the fabrication of an endothelialized hepatic tumor microtissue model based on the multicellular aggregates formed in the PLGA PMs, for applications in drug screening (**Figure** **1**). Initially, the PLGA‐based porous microarchitectures were fabricated using the microfluidic technology. Then, the formation of aggregated cell‐laden architectures, as well as endothelialized tumor microtissues were explored using the dynamic culture method by populating HepG2 cells and HUVECs in the PLGA PMs. Further, the engineered multicellular tumor microtissue model was employed to assess the cell responses of chemotherapeutic anticancer drugs, doxorubicin (DOX) and cisplatin (CIS). Finally, the cellular internalization of DOX and apoptotic events were evaluated.

## 2 Results and Discussions

### 2.1 Fabrication and Characterizations of PLGA PMs

**Figure 2:**
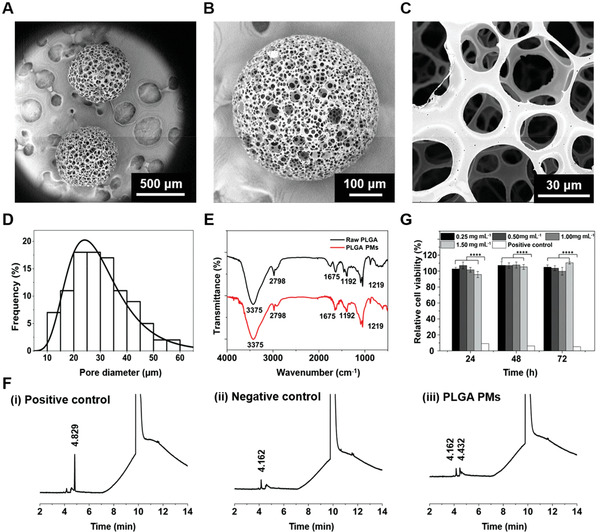
Fabrication and characterizations of PLGA PMs. A–C) SEM images of the size distribution and the morphology of the PLGA PMs. D,E) Pore size distribution and FTIR analysis of the fabricated PLGA PMs. F) Analysis of organic solvent residues by GC. The solvents pure DMF, as well as a mixture of DCM in DMF (0.0045%, v/v), were used as negative and positive controls, respectively. G) Cytotoxicity test in vitro showing the viabilities of HepG2 cells after incubation with different concentrations of PLGA PMs leach solution (0.25, 0.50, 1.00, and 1.50 mg mL^−1^) at different exposure times (24, 48, and 72 h). The medium containing phenol (0.64%, v/v) was used as the positive control. ::::*P* < 0.0001.

In the microfluidics‐assisted fabrication of PLGA PMs, the formation of uniform‐sized, discrete, and highly porous microarchitectures, as well as the parameter optimizations, were explicitly demonstrated in our previous report.^[^ ^11^ ^]^ Briefly, the removal of the uniformly distributed aqueous gelatin droplets led to the formation of porous structures. Accordingly, the PLGA PMs with interconnecting windows were prepared at the parameters of W/O ratio of 1:2.4, gelatin concentration of 7.5% (w/v), PLGA concentration of 2% (w/v), and flow rates of the continuous phase and dispersion phase set at 2 and 0.05 mL min^−1^, respectively. From the scanning electron microscopy (SEM) observations, it was evident that the uniform PLGA PMs with the average particle size of 395 µm (calculated by analyzing 100 randomly selected particles) were produced (**Figure** **2**A). The fabricated globular PMs possessed highly porous architectures with open and interconnecting windows (Figure 2B,C), in the pore size distribution range of 10–60 µm (Figure 2D), which would be substantially conducive for entry and distribution of cells in the interiors of PLGA PMs.

Further, the chemical functionalities of PLGA PMs were characterized using Fourier‐transform infrared (FTIR) spectroscopy and compared with the raw PLGA (Figure 2E). The peaks at 1675 and 2798 cm^−1^ could be attributed to C=O and C—H stretching vibrations, respectively. The peaks at around 1192 and 1219 cm^−1^ could be ascribed to the stretching vibrations of C—O—C. Moreover, the residual amount of dichloromethane (DCM) in the microarchitectures was examined to ensure the compatibility of the PLGA PMs. From the gas chromatograph (GC) recordings, the retention times (*t*_R_) of positive (DCM in dimethylformamide (DMF)), and negative (pure DMF) controls were ≈4.829 and 4.162 min, respectively, while the PLGA PMs resulted in 4.162 and 4.432 min, indicating that no DCM residues were observed in the PLGA PMs (Figure 2F). Although the biocompatibility of PLGA PMs was comprehensively investigated in our previous study,^[^ ^11^ ^]^ the cytocompatibility of PLGA PMs with HepG2 cells was still performed using the cell counting kit (CCK)‐8 assay. As depicted in Figure 2G, the viabilities of HepG2 cells at various concentrations of leach solutions of PLGA PMs were maintained at above 80% in all the treatments after 24, 48, and 72 h. Together, the results demonstrated that these PLGA‐based microarchitectures were highly compatible with HepG2 cells.^[^ ^11^ ^]^

### 2.2 Fabrication and Characterizations of Cell‐Laden PLGA PMs

**Figure 3:**
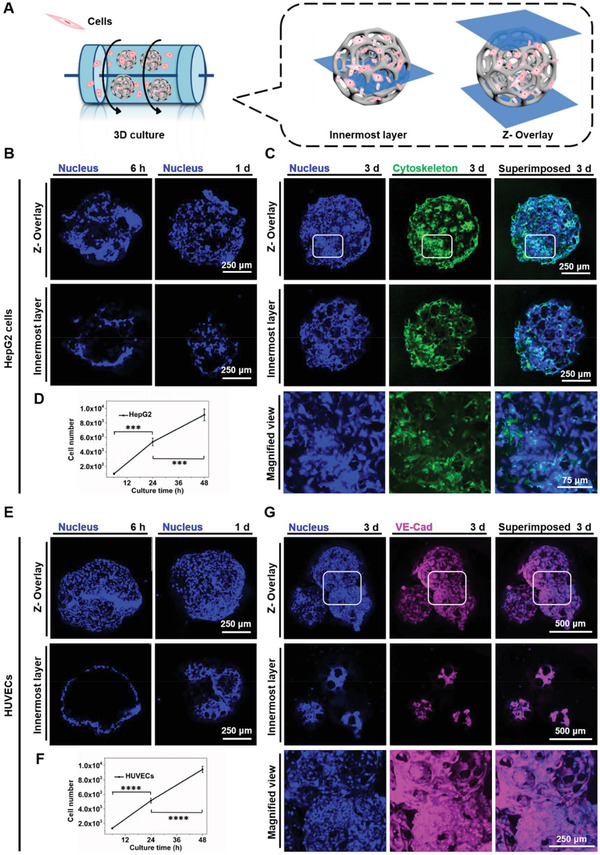
Construction of HepG2 cells and HUVECs‐laden PLGA PMs, respectively. A) The dynamic culture method for culture of PLGA PMs laden with cells. The dynamic culture was carried out at 37 °C and 110 rpm. B) CLSM images showing the adhesion of HepG2 cells cultured with the PLGA PMs in the dynamic culture method for various time periods (6 h and 1 d). C) CLSM images showing the cytoskeleton of HepG2 cells on the PLGA PMs skeleton after 3 d. D) Graphical representations showing adhesion and initial proliferation of the HepG2 cells (6, 24, and 48 h). E) CLSM images showing adhesion of HUVECs dynamic cultured with the PLGA PMs for various time periods (6 h and 1 d). F) Graphical representations showing adhesion and initial proliferation of HUVECs (6, 24, and 48 h). G) CLSM images showing the endothelial‐specific junction protein of HUVECs on the PLGA PMs skeleton after 3 d. :::*P* < 0.001 and ::::*P* < 0.0001.

Prior to the construction of the multicellular aggregates, the adhesion behaviors of both cell types, HepG2 cells, and HUVECs, on the PLGA PMs, were separately investigated. The dynamic culture approach was employed by incubating the PLGA PMs with the cell suspension in the culture medium (**Figure** **3**A). The distribution of the cells (nuclei counterstained with 4’,6‐diamidino‐2‐phenylindole (DAPI)), as well as human vascular endothelial‐cadherin (VE‐Cad) expression of HUVECs and cytoskeleton of HepG2 cells in the PLGA PMs were observed under a confocal laser scanning microscope (CLSM). As depicted in Figure 3B, it was observed that the fluorescence was distributed predominantly in the peripheries of the samples in the initial 6 h, signifying the adhesion of HepG2 cells to the surface of the PLGA architectures. Further, the fluorescence levels indicating the density of HepG2 cells in the PLGA PMs were augmented with the increase of the culture time, attributing to the highly open porous architectures facilitating adhesion as well as substantial infiltration of cells in the interior of PLGA PMs. In addition, the number of the HepG2 cells was visibly increased after 3 d. Moreover, the phalloidin‐stained cells depicting the morphological attributes of cells showed the vibrant cytoskeletal organization of HepG2 cells (Figure 3C).^[^ ^19^ ^]^ To further analyze the proliferation of HepG2 cells on the PLGA PMs, the cell number on the individual microsphere was recorded pertaining to the culture time (Figure 3D). The number of HepG2 cells on the PLGA PMs significantly increased over time after the initial adhesion. Notably, the number of cells per microsphere increased gradually from an average of 5370 cells in the initial 24 h to ≈9100 cells after 48 h in the dynamic culture.

Compared to the HepG2 cells, the HUVECs showed similar growth trends in terms of proliferation and infiltration into the interiors of the PLGA PMs in 24 h (Figure 3E). In addition, a significant increase in the number of HUVECs on the PLGA PMs was also observed after initial adhesion within the polymeric architectures (Figure 3F). As revealed by CLSM observations, the expression of endothelium‐specific junction biomarker, VE‐Cad on their membranes was profoundly evident (Figure 3G).^[^ ^20^ ^]^ Further, with the increase in culture time, the discrete PLGA PMs self‐assembled, resulting in the aggregated constructs of HUVECs‐laden microspheres through establishing intercellular interactions and substantial binding of the PLGA PMs. Plausibly, the good adhesion and interactions between the cells, as well as the high expression of VE‐Cad of HUVECs might have contributed to the formation of HUVECs‐laden multi‐PM agglomerates.^[^ ^21^ ^]^ These findings suggested that the PLGA PMs were compatible with different cell types, which would be more conducive for further formation of the tumor microtissues through the interactions of multicellular aggregates within individual PLGA microarchitectures.

### 2.3 Formation of Tumor Microtissues and Bioefficacy Assessments

**Figure 4:**
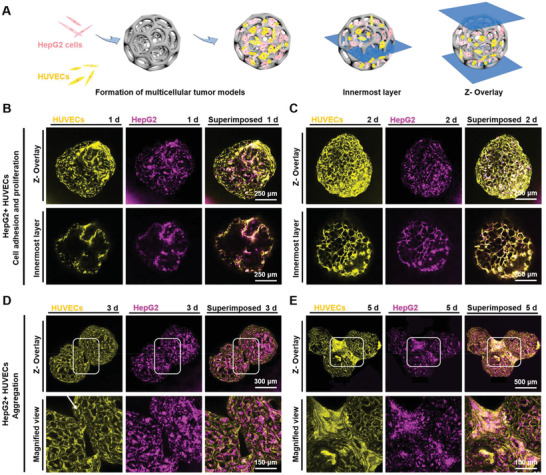
Coculture of HepG2 cells and HUVECs in PLGA PMs. A) The dynamic culture method for coculture of HepG2 cells and HUVECs. B–E) Fluorescence microscopy images showing the adhesion and distributions of HepG2 cells (purple) and HUVECs (yellow) on the PLGA PMs after 1, 2, 3, and 5 d of coculture.

In the native tumor tissues, the complex composition involving multiple cell types with heterotypic cell–cell interactions can substantially regulate the growth, invasion, and metastasis of tumor cells.^[^ ^13^, ^22^ ^]^ Considering the role of vascularization in the tumor growth in vivo, HUVECs were employed along with HepG2 cells to mimic the complex liver tumor microenvironment and tumor amplification through multicellular adhesion, growth, as well as aggregation of PLGA PMs‐based architectures.^[^ ^14^, ^23^ ^]^ HepG2 and HUVEC suspensions were labeled with different cell trackers for their visibility in PLGA PMs (**Figure** **4**A). After 1 d of dynamic culture, HepG2 cells and HUVECs possessed surface areas similar to HepG2 cells alone on PLGA PMs, and they filled the spaces in the interiors of PLGA PMs after 2 d (Figure 4B,C). Moreover, a few of the HepG2 cells overlapped with the HUVECs. Further, the aggregation of multiple PLGA PMs was achieved, resulting in tight cell–cell interactions between the individual PMs (Figure 4D). Notably, the bridging between the PLGA PMs appeared more evident after 5 d of incubation, thus providing enriched adhesion sites of cells due to strong cell–cell interactions (Figure 4E). Together, these experimental results indicated that the tight cell–cell junctions between the individual PLGA PM played a crucial role in the construction and dimensional control of the aggregated tumor microtissues.

**Figure 5:**
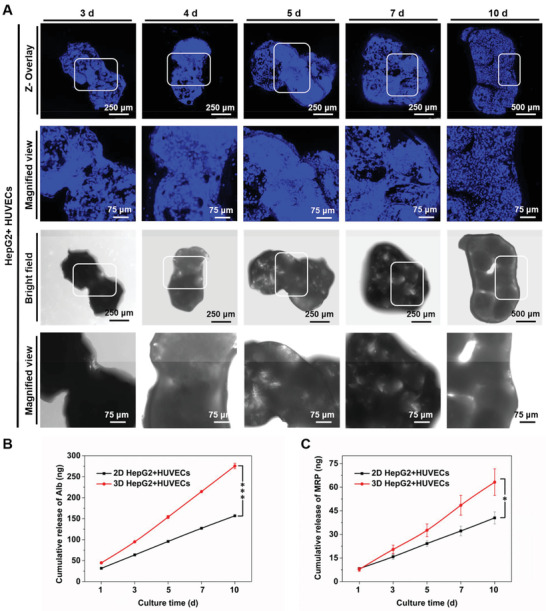
Evolution of multicellular tumor microtissue formation. A) Fluorescence and bright‐field microscopy images showing the growth of HepG2 cells and HUVECs on the PLGA PMs after 3, 4, 5, 7, and 10 d of coculture. The connections between the cell‐laden PLGA PMs were observed after 3 d (white squares), and the aggregation of these PLGA architectures was achieved with prolonged culture time. B) The release levels of Alb measured by ELISA. C) The release levels of MRP measured by ELISA. :*P* < 0.05 and :::*P* < 0.001.

Since the fluorescence of the cell trackers would be more prone to quenching with prolonged culture time, the nuclei were further stained by DAPI in the dynamic culture of HepG2 cells and HUVECs to validate the aggregation trend of the multiple cell‐laden PLGA PMs (**Figure** **5**A). It was observed that the similar cell bridging appeared at 3 d (white squares), which strengthened the cellular interactions between the individual multicellular aggregates with the increase in culture time. Notably, when the culture time reached 10 d, the multicellular aggregates gathered into a larger mass with dimensions of ≈1 mm × 2 mm (width × length), possibly attributing to the enhanced communication between the cells in PLGA PMs. The stability of the tumor microtissues was examined using a papillon dropper. Meanwhile, the HepG2/HUVEC aggregates were constructed without PLGA PMs to serve as a control, using the dynamic culture method (Video S1, Supporting Information). In the case of the same culture time, the HepG2/HUVECs‐laden PLGA PMs clearly agglomerated into a larger mass and had no apparent shedding compared with the nonscaffolding group, revealing the significance of the PLGA PMs‐porous architectures toward the stable assembly of cells as well as substantial tumor growth.^[^ ^24^ ^]^ To further examine the tight junctions between the PMs and rapid growth of the tumor microtissues, the culture time was prolonged to 15 d for HepG2/HUVECs‐laden PLGA PMs. It was noted that no signs of separation of PLGA PMs were observed after several times of blowing (Video S2, Supporting Information). Together, the culturing of HepG2/HUVECs with the PLGA PMs resulted in a level of tissue complexity with the established cell–cell communications and a similar structure of the hepatic lobules, indicating the construction of the microenvironment of hepatic tumors.^[^ ^16^ ^]^

To further demonstrate the bioefficacy of the tumor microtissue, the levels of albumin (Alb), an essential protein secreted by the liver, and multidrug resistance‐associated protein (MRP), one of the multidrug resistance‐formation proteins released by HepG2 cells, were measured.^[^ ^25^ ^]^ The standard curves of Alb protein and MRP are presented in Figure S1 (Supporting Information). As shown in Figure 5B,C, significant differences in the secretions of Alb and MRP by HepG2 cells were observed between the static 2D and dynamic 3D culture methods. Increased secretion of Alb might have contributed to the aggregation of PLGA architectures due to the viscous and glial properties of Alb.^[^ ^26^ ^]^ Similarly, the levels of MRP were also augmented, which participates in the regulation of redistributing intracellular constituents to protect the cells.^[^ ^26^ ^]^ It is believed that the adherence of individual architectures and aggregation of multicellular‐laden PLGA PMs could to a good extent represent the vascularized tumor microtissues in vitro.

### 2.4 Drug Screening

**Figure 6:**
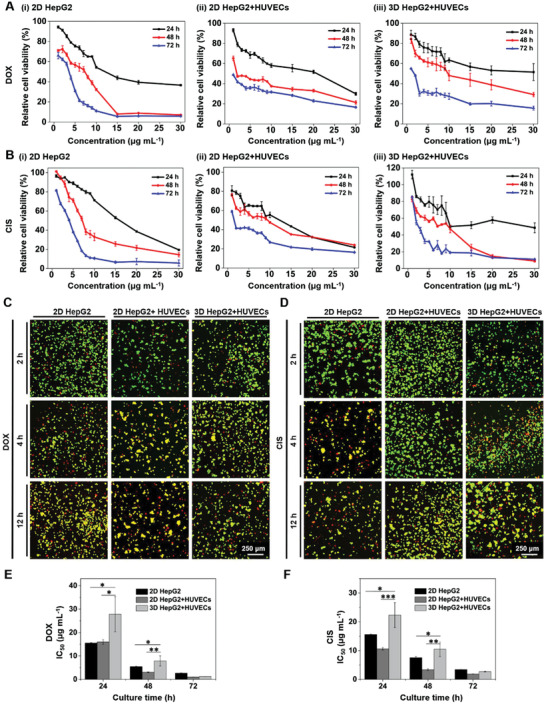
Drug evaluation on the multicellular tumor microtissue model. A,B) Cytotoxicity tests in vitro showing the relative viabilities of cells after incubation with different concentrations of A) DOX and B) CIS. C,D) Fluorescence microscopy images showing AO/EB staining of 2D‐ and 3D‐cultured cells at different time points (2, 4, and 12 h). The live and dead cells were stained in green and red, respectively. The cells in yellow represent the early stage of apoptosis after incubation with drugs. E,F) Dose‐responses of 2D‐ and 3D‐cultured HepG2 cells and HUVECs to E) DOX and F) CIS. The concentrations of DOX and CIS used were both 15 µg mL^−1^. :*P* < 0.05, ::*P* < 0.01, and :::*P* < 0.001.

During preclinical screening of drugs, the cytotoxicity assays are primarily executed using various cell lines to predict the effect of drug molecules on cells.^[^ ^6^, ^27^ ^]^ As commonly used chemotherapeutic agents, DOX and CIS,^[^ ^28^ ^]^ were considered for evaluating the application in drug screening of our PLGA PMs‐based hepatic tumor microtissue model.^[^ ^29^ ^]^ Initially, to ensure the effects of chemotherapeutic drugs, the cell number of HepG2 cells was assessed after separately exposing to DOX and CIS at a concentration range of 1–30 µg mL^−1^ using the CCK‐8 assay (**Figure** **6**A‐(i)).^[^ ^30^ ^]^ Then, the sensitivities of HepG2 cells and HUVECs to drugs were analyzed under 2D static and 3D dynamic culture conditions. As depicted in Figure 6A‐(ii) and (iii), the viabilities of the different cell types were decreased with the increase in DOX concentration and exposure time. The same trend was also observed for CIS (Figure 6B). Further, the live/dead analyses of HepG2/HUVECs in 2D plate and 3D dynamic culture methods were conducted using acridine orange/ethidium bromide (AO/EB) staining (Figure 6C,D). After incubation with DOX (15 µg mL^−1^) or CIS (15 µg mL^−1^) for different time intervals (2, 4, and 12 h), dead cells (red fluorescence) were apparent in the treatment group. Meanwhile, a higher number of cells entered the early apoptotic phase (yellow fluorescence) and more dead cells were evident (red fluorescence) after 12 h of incubation compared to preceding time points, indicating that the AO/EB staining results were consistent with the cytotoxicity analysis.

The half‐maximal inhibitory concentration (IC_50_) values of DOX were further analyzed in all groups based on the cytotoxicity results (Figure 6E). The IC_50_ values for 2D‐ and 3D‐cultured HepG2/HUVECs were 15.94 ± 0.98 and 27.80 ± 7.53 µg mL^−1^, respectively, at 24 h of incubation, and 3.07 ± 0.16 and 7.87 ± 2.16 µg mL^−1^, respectively, at 48 h. The high density of the cells packed in the PLGA PM‐based tumor microtissues might have led to the difference in the diffusion and eventual availability of the drugs compared to the 2D monolayers. In addition, the cells in the PLGA PMs might have resulted in the formation of a highly dense ECM microenvironment, further leading to an increase in the IC_50_ values.^[^ ^24^, ^31^ ^]^ After incubation for 72 h with DOX, no significant differences in the IC_50_ values of any of the treatment groups were observed. Furthermore, we substituted the DOX with CIS and repeated the investigations and familiar results were observed (Figure 6F). These experimental results indicated that the drug assessments with various drug types could be effectively conducted.

To demonstrate the feasibility of accommodating various other cell types, we further cocultured HepG2 cells and fibroblasts (L929) for the construction of the multicellular tumor microtissue model. The adhesion and growth of HepG2 and L929 cells using dynamic culture on PLGA PMs were investigated in vitro (Figures S2–S4, Supporting Information). Similar trends of changes in the IC_50_ values were observed in the culture of HepG2/L929 cells using 2D and 3D culture methods. We believe that different cell types could be conveniently selected as parenchymal and nonparenchymal cells to model the desired tumor microenvironments using our PLGA PM‐based modular microtissue construction strategy.

### 2.5 Cellular Internalization Efficiency

**Figure 7:**
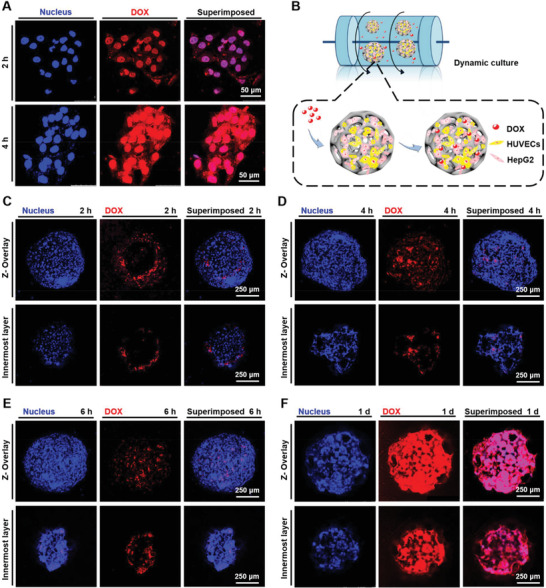
Cellular internalization of DOX (red) along with DAPI staining for the nuclei (blue). The concentration of DOX was 15 µg mL^−1^. A) DOX internalization by 2D‐cocultured HepG2 cells and HUVECs at different time points (2 and 4 h). DOX was internalized into the cells starting by 2 h, and a significant amount of DOX was accumulated in the cells at 4 h. B) DOX internalization by cells in the multicellular aggregates (HepG2 cells and HUVECs) under 3D dynamic culture. The dynamic culture was carried out at 37 °C and 110 rpm. C–F) Fluorescence microscopy images showing cellular internalization of DOX in the multicellular aggregates (HepG2 cells and HUVECs) at different time points (2, 4, and 6 h, as well as 1 d). Limited amounts of DOX were observed around the peripheries of the individual microspheres after incubation for the same time with the 2D culture (2 and 4 h). The amount of DOX gradually increased after 1 d of incubation.

To further confirm the internalization efficiency of cells in the PLGA PMs, the autofluorescent DOX molecules were tracked in the 2D culture and 3D multicellular aggregates using CLSM.^[^ ^32^ ^]^ For 2D HepG2/HUVECs coculture (**Figure** **7**A), the DOX molecules were localized in the cells after 2 h of incubation, and significant amounts of DOX were accumulated after 4 h. These short incubation times were chosen to avoid the interferences caused by cell detachment and DOX‐induced cell death.^[^ ^30^ ^]^ Contrarily, it was evident that limited amounts of DOX were observed around the peripheries of the PLGA PMs‐based 3D tumor microtissue model after incubation for the same time with the 2D culture (Figure 7B). Interestingly, with the prolonged incubation period, the red fluorescence of DOX gradually increased after 1 d, attributing to the slow diffusion and infiltration of DOX molecules due to the high density of cells in the multicellular aggregates (Figure 7C–F).

**Figure 8:**
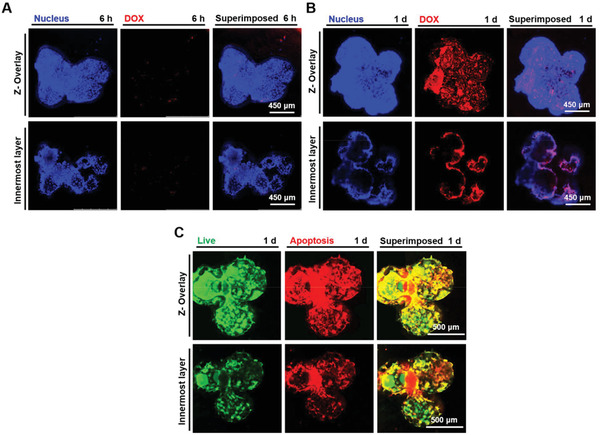
Cellular internalization of DOX in the multicellular tumor microtissues. A,B) Fluorescence microscopy images showing cellular internalization of DOX (red) at 6 h and 1 d along with DAPI staining for the nuclei (blue). A minimal amount of DOX was observed after 6 h of incubation. The distribution of red fluorescence suggested that the DOX was mainly internalized in the peripheries of the tumor microtissues. C) Apoptosis analysis of cells in the multicellular tumor microtissues at 1 d of DOX treatment (green: live cells; red: dead and late‐apoptotic cells; yellow: apoptotic cells). The cells with low viability were mainly distributed in the peripheries of the microtissues.

Furthermore, to validate the formation of tight junctions between the individual multicellular aggregates and internalization efficiency of the DOX molecules, CLSM observations of the tumor microtissues were recorded. Similar trends of delayed internalization in the individual multicellular aggregates were attained in the tumor microtissues. It was evident that a minimal amount of DOX appeared around the aggregations of cell‐laden PLGA PMs after 6 h of incubation (**Figure** **8**A), which however, resulted in enriched internalization of DOX with prolonged incubation time to 1 d (Figure 8B). Further, cell apoptosis was investigated using annexin staining after 24 h of incubation with DOX (Figure 8C). It was observed that the apoptotic cells (yellow fluorescence) were apparent on the peripheries of the tumor microtissues, while the cells in the interiors of the aggregates remained active (green fluorescence) after 1 d of DOX treatment, attributing to the difference in the availability of the drug in the constructed tumor microtissues. The low cell viability on the peripheries of the microtissue might be attributed to the accumulation of DOX, which was consistent with the cellular internalization analysis of the DOX. Together, we believe that the delayed infiltration of drugs in the constructed 3D multicellular tumor microtissues might have influenced the overall performance, including inhibitory effects as well as pharmacological attributes of the drugs. Meanwhile, the 3D multicellular tumor microtissue model provides an engineering advance that to a good extent mimics tight vascularized organization and aggregation in vitro, which may find potential use in drug screening and cancer research in the future.

## 3 Conclusions

In summary, we have reported the construction of a 3D liver tumor microtissue model using PLGA PMs co‐laden with HUVECs/HepG2 cells for potential applications in drug screening. HUVECs and HepG2 were cocultured within PLGA PMs such that they would facilitate the connection and aggregation of PLGA PMs and form a physiologically relevant tumor microenvironment in vitro. Furthermore, it was found that the drug evaluation capability of the architectures was significantly enhanced compared to the 2D plate culture method while using DOX and CIS as model drugs. The enhancement of drug evaluation was not only manifested in the ability of the tumor microtissue model to significantly prolong the time of internalization of the drug molecules into the interior, but also in the different availability of the drugs for the cells in the different locations. However, in‐depth analyses are still required to demonstrate the explicit mechanism of drug effects and metabolism. Together, the construction of a multicellular hepatic tumor microtissue model based on PLGA PMs co‐laden with HUVECs and HepG2 cells is potentially of significance for promoting drug screening in vitro, thus providing an enabling platform for drug development and cancer therapy.

## 4 Experimental Section

##### Materials

PLGA (66–107 kDa, lactide:glycolide 75:25), gelatin (from porcine skin, Type A), and polyvinyl alcohol (PVA) were obtained from Sigma‐Aldrich (St. Louis). HepG2 cells, HUVECs, DAPI, L929, phosphate‐buffered saline (PBS), CCK‐8 working solution, annexin V‐fluorescein isothiocyanate isomer (FITC) reagent, and AO/EB were purchased from Keygen Biotech Co., Ltd (Nanjing, China). Trypsin was purchased from Amresco (Solon). DCM, ethylenediaminetetraacetic acid (EDTA), and DMF were purchased from Sinopharm Chemical Reagent Co., Ltd. (Shanghai). Fetal bovine serum (FBS), endothelial basal medium (F‐12K), penicillin‐streptomycin, and Dulbecco's modified Eagle medium (DMEM) were purchased from Gibco (Grand Island). DOX and Triton X‐100 were obtained from Aladdin Industrial Inc. (Shanghai, China). CIS was obtained from Meryer Chemical Technology Co., Ltd. (Shanghai). Paraformaldehyde (4%, w/v) and FITC‐phalloidin were purchased from Solarbio Life Sciences (Beijing). VE‐Cad primary polyclonal antibody, goat antirabbit IgG (H+L) secondary antibody, and cell labeling kits (Qtracker 525 and 655) were obtained from Thermo Fisher (Waltham).

##### Preparation of PLGA PMs

Uniform‐sized PLGA PMs were fabricated using the microfluidic approach, as reported in the previous study.^[^ ^11^ ^]^ Briefly, a customized coaxial microfluidic system made up of plastic tubes (inner diameter, ID: 1 mm), a glass capillary (outer diameter, OD: 1 mm), and a needle (27G, ID: 210 µm, OD: 400 µm) were used. The aqueous solution of gelatin (1 mL, 7.5%, w/v) was added to the DCM solution of PLGA (2.4 mL, 2%, w/v) as the internal water phase. The ultrasonic emulsification (Scientz, Ningbo) time was set as 90 s (ultrasonic treatment 1 s, interval 2 s) for stabilization of ultrasonic emulsification. Further, the dispersion phase introduced into the continuous PVA (1%, w/v) aqueous solution. The flow rates of continuous (PVA solution) and dispersion (PLGA emulsion) phases were set at 2 and 0.05 mL min^−1^, respectively. The gelatin in the samples was then removed by exposing the microspheres to deionized water at 45 °C for 1 h. Finally, the samples were lyophilized overnight.

##### Residue of DCM

The DCM residue in the PLGA PMs was detected by a headspace sampler (G1888, Agilent Technologies, Santa Clara) coupled to a GC interfaced with a flame ionization detector (6890N, Agilent Technologies). The solvent mixture of DCM and DMF (0.0045%, v/v), as well as DMF, were used as positive and negative controls, respectively. The 100 mg of PLGA PMs was dissolved in 5 mL of DMF and then placed in the stoppered glass bottle. The parameters of the chromatographic system were set as follows: carrier gas: nitrogen (N_2_; flow rate: 1.0 mL min^−1^; distributary mode at 20:1 flow ratio); inlet temperature = 200 °C; column temperature range = 38 to 230 °C; bottle heating temperature = 80 °C; bottle heating time = 30 min; heating rate = 20 °C min^−1^; and detector temperature = 250 °C.

##### FTIR

The chemical functionalities of materials were analyzed by FTIR spectroscope using the potassium bromide (KBr, Fisher Scientific Ltd., Loughborough) pellet method. The raw PLGA and PLGA PMs were grounded with KBr separately. The wavenumber region of spectra was 4000–400 cm^−1^.

##### Biocompatibility Assessment

Prior to the coculture of the cells and PLGA PMs, the cytotoxicity in vitro of these samples was investigated. HepG2 cells were cultured in DMEM supplemented with FBS (10%, v/v) and penicillin‐streptomycin (1%, v/v) and incubated at 37 °C in 5% CO_2_ and 95% relative humidity. HUVECs were cultured in F‐12K medium supplemented with penicillin‐streptomycin (1%, v/v), FBS (10%, v/v), and 0.05 mg mL^−1^ of endothelial cell growth supplement (ECGS, Invitrogen, Carlsbad), and incubated at 37 °C in 5% CO_2_ and 95% relative humidity. The cytotoxicity of PLGA PMs was analyzed using the CCK‐8 kit.^[^ ^33^ ^]^ Briefly, HepG2 cells were seeded in a 96‐well plate at a density of 5 × 10^3^ cells per well. Then, the cells were incubated with the leach solution of the PLGA PMs at different concentrations (0.25, 0.5, 1.0, and 1.5 mg mL^−1^) for 24, 48, and 72 h. DMEM containing phenol (0.64%, v/v) and pure DMEM were used as the positive and negative controls, respectively. After incubation for predetermined intervals, the CCK‐8 (10 µL of CCK‐8 in 100 µL of fresh medium) working solution was added to each well and incubated at 37 °C further for 2 h. The absorbance values at 450 nm were recorded using a microplate reader (Thermo Fisher). The relative growth rate (RGR) was calculated as below

(1) $$\begin{array}{ccc} \mathrm{RGR}\% & = & \left(\mathrm{sample}\;\mathrm{group}\;OD_{450\;\mathrm{nm}}-\mathrm{blank}\;\mathrm{group}\;OD_{450\;\mathrm{nm}}\right)/\\ & & \left(\mathrm{positive}\;\mathrm{control}\;\mathrm{group}\;OD_{450\;\mathrm{nm}}-\mathrm{blank}\;\mathrm{group}\;OD_{450\;\mathrm{nm}}\right)\\ & & \times \;100 \end{array}$$

##### Cell Adhesion on PLGA PMs

For cell adhesion on PLGA PMs, 3 mg of PLGA PMs were cultured with cell suspension (5 mL, 2 × 10^6^ cells mL^−1^) in a centrifuge tube (50 mL) under dynamic culture (37 °C and 110 rpm), as reported previously.^[^ ^11^ ^]^ Prior to the construction of the multicellular aggregates, the adhesion efficiencies of HepG2 cells and HUVECs to the PLGA PMs were investigated separately using the nuclei staining and cell counting methods. For nuclei staining, the cell‐laden PLGA PMs were washed thrice with PBS after 6, 24, and 72 h of incubation. The cell‐laden PMs were then fixed with paraformaldehyde (4%, w/v) for 1 h and then soaked in DAPI for 15 min. The images were captured using a CLSM (Leica TCS SP8, Wetzlar, Germany). Notably, the 3D scanning was used to capture the images to demonstrate the adhesion and distribution of cells in the inner cavities of PLGA PMs. Meanwhile, the cell counts on the PLGA PMs were measured using the hemocytometer after 6, 24, and 48 h of culture. The samples were incubated in trypsin (0.25%, w/v) with EDTA (0.05%, w/v) for 3 min. After termination of digestion with medium, the cell numbers were counted under the microscope (C‐35‐AD‐2, Olympus, Tokyo, Japan). Six parallel samples were set at each time point. Further, the construction of cell‐laden PLGA PMs was confirmed by immunofluorescence staining of cytoskeleton for HepG2‐laden PLGA PMs and VE‐Cad expression for HUVECs‐laden PLGA PMs along with DAPI for nuclei counterstaining. Briefly, the samples were fixed with paraformaldehyde (4%, w/v) for 1 h and then soaked in Triton X‐100 (0.1%, v/v) to permeabilize the cell membranes. For cytoskeleton staining, the samples were incubated with FITC‐phalloidin for 1 h and then transferred to DAPI solution after washing with PBS thrice. For VE‐Cad expression, the samples were soaked in bovine serum albumin (BSA, 10%, w/v, Keygen) in PBS to block nonspecific binding for 30 min. The VE‐Cad primary polyclonal antibody (1:50 dilution in PBS) was added and incubated for overnight at 4 °C. Further, the samples were then incubated with goat antirabbit IgG (H+L) secondary antibody (1:200 dilution in PBS) and incubated for 2 h at room temperature. Finally, the nuclei were labeled using DAPI for 20 min and the images were captured.

##### Construction of the Multicellular Aggregates and Tumor Microtissues

The PLGA PMs were cultured with cell suspensions of HepG2/HUVECs or HepG2/L929 cells. Prior to the fabrication, the cells were tracked separately by cell labeling kits for the observation of the cell distribution and proliferation. The HUVECs and L929 cells were labeled by the Qtracker 525 cell labeling kit, and the HepG2 cells were labeled by the Qtracker 655 cell labeling kit based on the manufacturer's instructions. Briefly, after cell digestion, the cells were incubated in the labeling kit for 50 min and washed twice with the growth medium. During the fabrication of the PLGA PMs‐based multicellular tumor model, the HepG2 cells and HUVECs at a ratio of 1:1, and the culture medium by mixing DMEM and F‐12K complete medium at a ratio of 1:1 was used. Additional assessments of cell proliferation and aggregation were made by nuclei staining images at predetermined time intervals using CLSM.

##### Specific Protein Releases from the Tumor Microtissues

The releases of various cell‐specific proteins, i.e., Alb and MRP of HepG2 cells using 2D plate culture and 3D dynamic culture, were analyzed using enzyme‐linked immunosorbent assay kits (ELISA, Thermo Fisher). For 2D culture, 1.5 × 10^4^ cells per well of HUVECs and 1.5 × 10^4^ cells per well of HepG2 cells were cocultured in a 6‐well plate and the final total cell number was set as 3 × 10^4^ cells per well. For 3D culture, HUVECs and HepG2 cells were mixed and cultured with PLGA PMs at approximately the same cell numbers according to results of cell counting. After 24 h, PLGA PMs were washed with PBS to remove the nonadherent cells, which was then replaced with the medium. For the ELISA assays, the culture medium was collected, stored at 4 °C, and then replaced with fresh culture medium at the specified time points. The concentrations of Alb and MRP in the supernatant were quantified following the manufacturer's instructions.

##### Cytotoxicity In Vitro

The influences of various chemotherapeutic drugs on cells in PLGA PMs were studied by taking DOX and CIS as model drugs. It should be noted that the number of cells on the 2D plate and 3D PLGA PMs were kept approximately the same. For 2D monolayer culture, the cells were seeded in a 96‐well plate at a density of 5 × 10^3^ cells per well. Specifically, for the multicellular aggregates, the cell‐laden PLGA PMs (one sample per well) were seeded in a 96‐well plate after 24 h of dynamic culture. Different concentrations of DOX and CIS (ranging from 0 to 30 µg mL^−1^) were separately added to the wells and incubated for 24, 48, and 72 h. The CCK‐8 assay was performed to measure the cell metabolic activities.

##### Cell Live/Dead Imaging

A kit with AO/EB dual stains was used to assess cell viability at different time points (2, 4, and 12 h) by following the manufacturer's instruction. Considering the IC_50_ values, the concentrations of DOX and CIS used in the treatment group were both 15 µg mL^−1^. For 2D monolayer culture, the cells were seeded in a 24‐well plate at a density of 3 × 10^4^ cells per well and then incubated with DOX and CIS. For the multicellular aggregates, the cell‐laden PLGA PMs (6 samples per well) were seeded in a 24‐well plate after 24 h of dynamic culture and incubated with DOX or CIS. After incubation, the supernatant and cell digestion were collected, and the cells were then stained after centrifugation (2000 rpm for 5 min).

##### Cellular Internalization of DOX and Apoptosis Analysis

The cellular internalization behaviors of DOX (15 µg mL^−1^) for 2D monolayer culture, multicellular aggregates, and tumor microtissues were analyzed by CLSM. DOX was visualized with its intrinsic fluorescence property. The nuclei were counterstained with DAPI. In addition, immunofluorescence staining of annexin V‐FITC was employed to demonstrate cell apoptosis.

##### Statistical Analysis

Data were presented as means ± standard deviations (SDs). The values of *P* < 0.05 were considered statistically significant.

## Conflict of Interest

The authors declare no conflict of interest.

## Supporting information

Supporting Information

Supplemental Video 1

Supplemental Video 2
