# Supplementary material for: Modeling Endothelialized Hepatic Tumor Microtissues for Drug Screening
Source: Adv Sci (Weinh). 2020 Sep 21;7(21):2002002. doi: 10.1002/advs.202002002 (PMC7610277; doi:10.1002/advs.202002002)
Supplement: Supplementary file 1 — Supporting Information [file ADVS-7-2002002-s001.pdf]

© 2020 Wiley-VCH GmbH

ADVANCED SCIENCE
Open Access

Supporting Information

for *Adv. Sci.*, DOI: 10.1002/advs.202002002

Modeling Endothelialized Hepatic Tumor Microtissues for Drug Screening

Ying Wang, Ranjith Kumar Kankala, Jianting Zhang, Liuzhi Hao, Kai Zhu, Shibin Wang, Yu Shrike Zhang, and Aizheng Chen**

Supporting information

Modeling Endothelialized Hepatic Tumor Microtissues for Drug Screening

Ying Wang, Ranjith Kumar Kankala, Jianting Zhang, Liuzhi Hao, Kai Zhu, Shibin Wang, Yu Shrike Zhang, * *Aizheng Chen* *

Y. Wang[+], Prof. R. K. Kankala[+], J. Zhang, L. Hao, Prof. S. Wang, Prof. A. Chen
Institute of Biomaterials and Tissue Engineering
Huaqiao University
Xiamen 361021, P. R. China
Email: azchen@hqu.edu.cn

Prof. R. K. Kankala, J. Zhang, Prof. S. Wang, Prof. A. Chen
Fujian Provincial Key Laboratory of Biochemical Technology
Huaqiao University
Xiamen 361021, P. R. China

Prof. K. Zhu
Department of Cardiac Surgery
Zhongshan Hospital
Fudan University
Shanghai 200032, P. R. China

Prof. Y. S. Zhang
Division of Engineering in Medicine, Brigham and Women's Hospital
Department of Medicine, Harvard Medical School
Cambridge, MA 02139, USA
Email: yszhang@research.bwh.harvard.edu

[+]These authors contributed equally to this work.

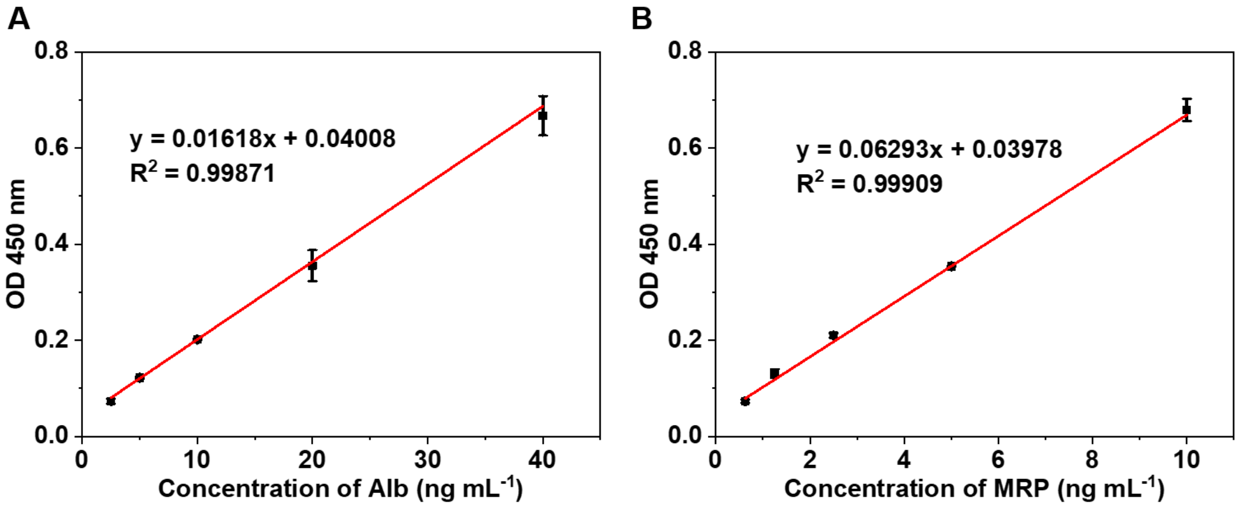

Figure S1. Standard curves of (A) Alb and (B) MRP measured by ELISA.

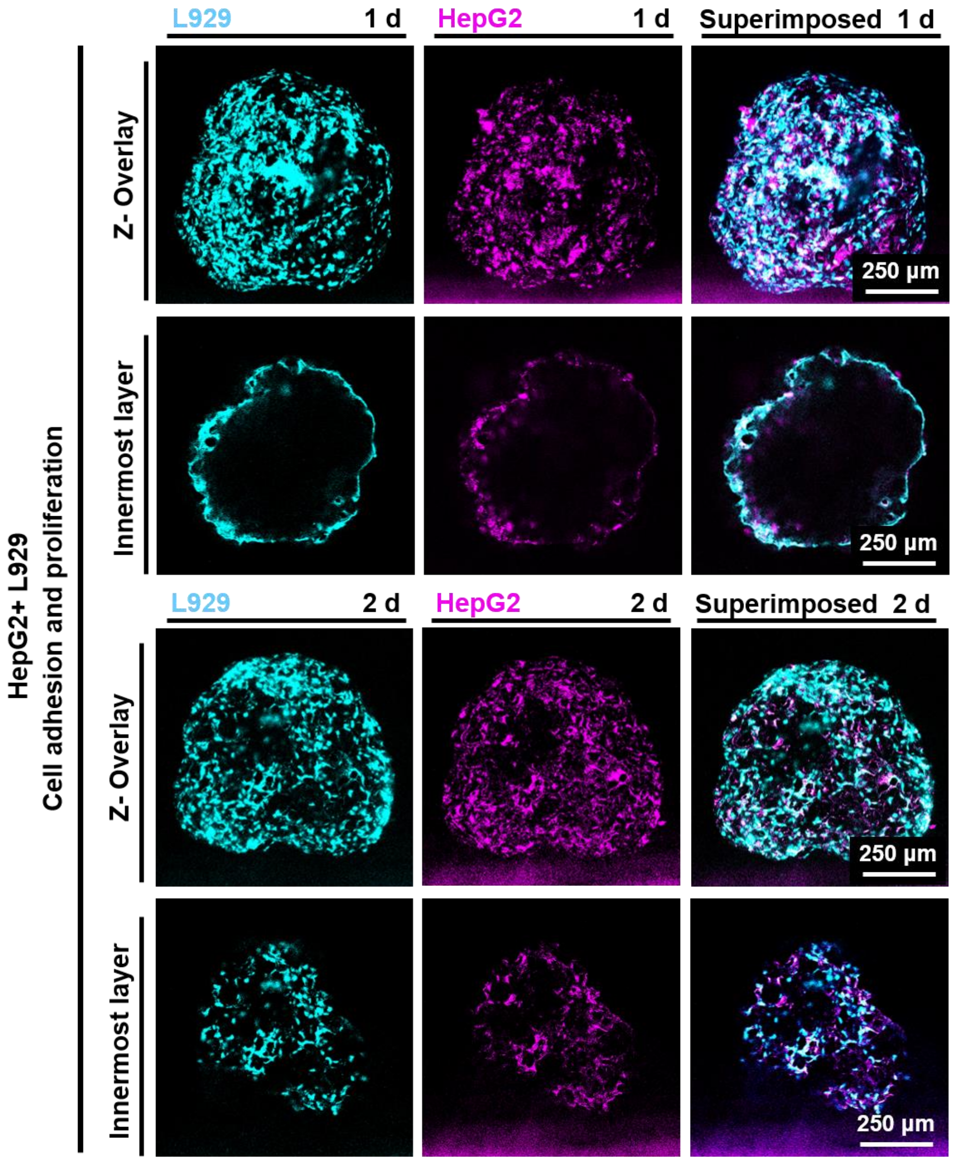

Figure S2. Coculture of HepG2 cells and L929 cells and study of their colocalization. Fluorescence microscopy images showing the adhesion and distributions of HepG2 (purple) and L929 (blue) cells on the PLGA PMs after 1 and 2 d of coculture.

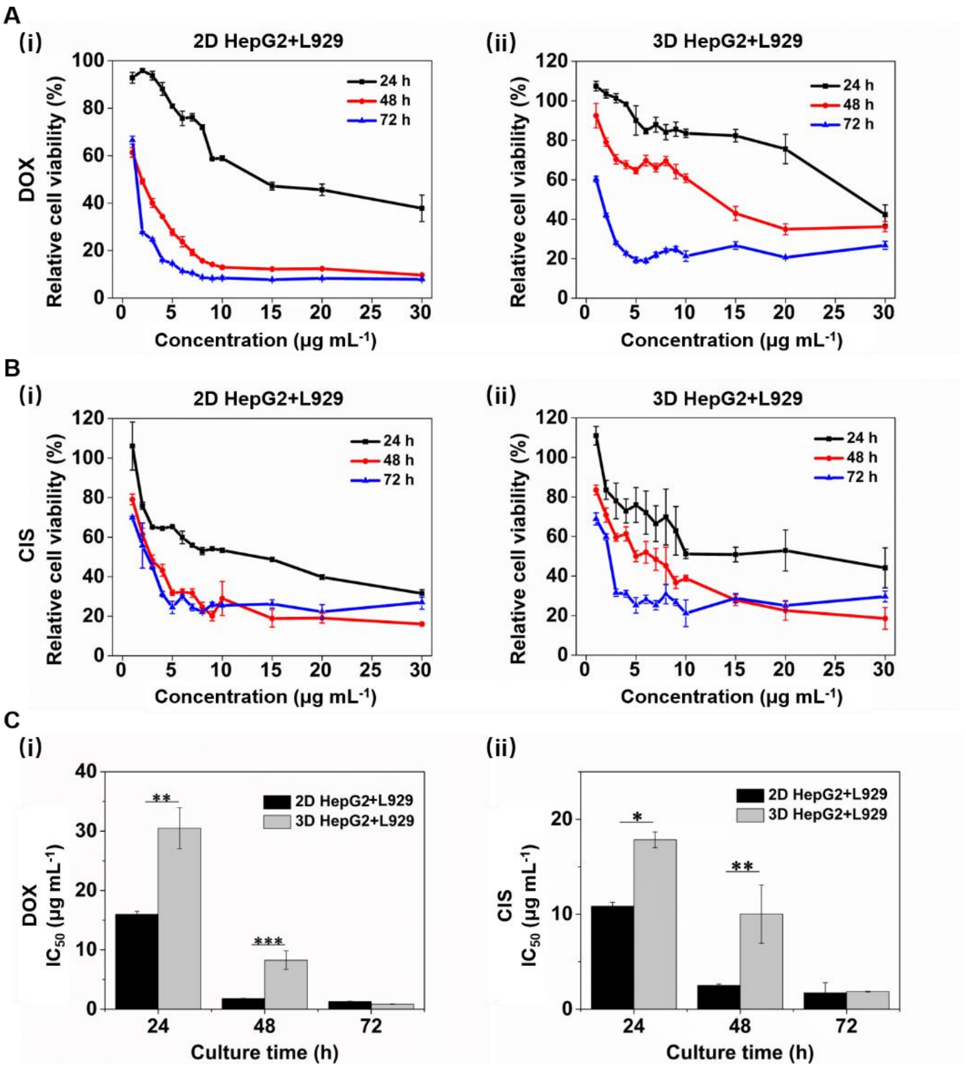

Figure S3. Drug evaluation on the multicellular tumor microtissue model using coculture of HepG2 and L929 cells. (A) *In vitro* cytotoxicity tests showing the relative viabilities of cells after treatment with different concentrations of DOX for 24, 48, and 72 h. (B) *In vitro* cytotoxicity tests showing the relative viabilities of cells after treatment with different concentrations of CIS for 24, 48, and 72 h. (C) Dose-responses of 2D- and 3D-cultured HepG2 cells and L929 cells to DOX and CIS, respectively. The concentrations of DOX and CIS used were both 15 μg mL^{-1}. *$P < 0.05$, **$P < 0.01$, and ***$P < 0.001$.

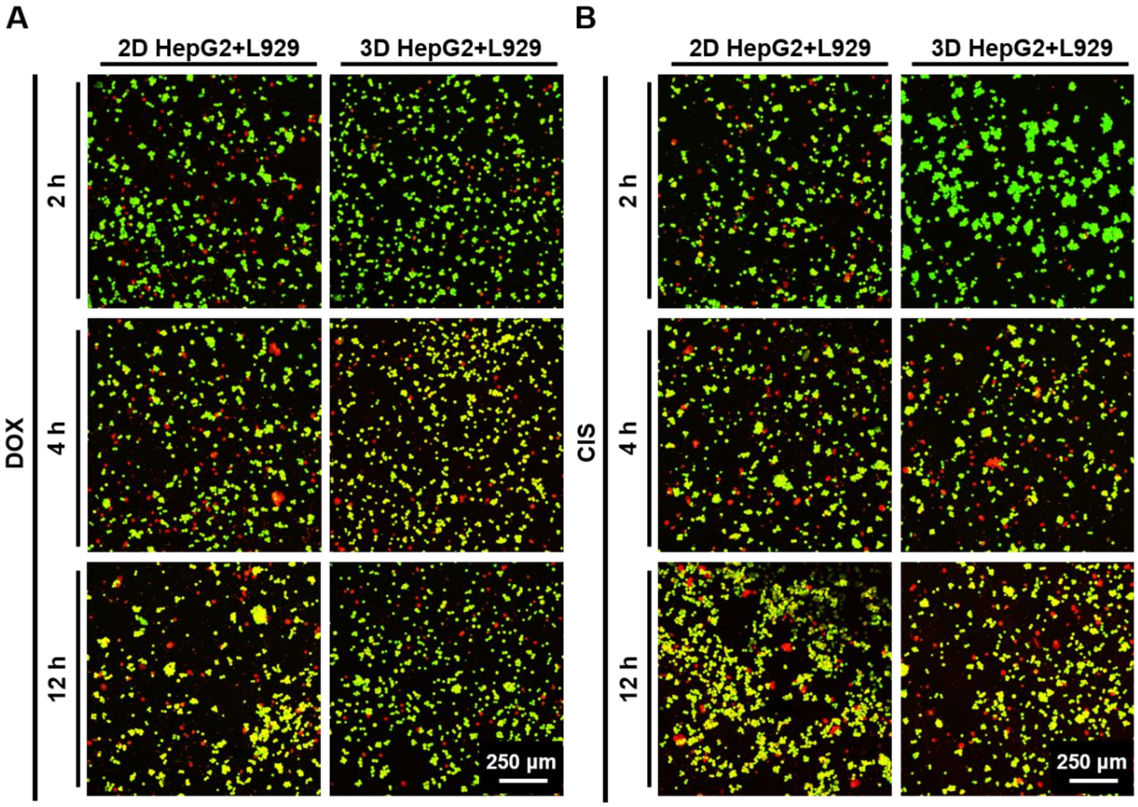

Figure S4. Fluorescence microscopy images showing of 2D- and 3D-cocultured HepG2 and L929 cells at different time points (2, 4, and 12 h). The live and dead cells were stained in green and red, respectively. The cells in yellow are at the early stage of apoptosis for cells after different times of incubation with DOX.
